# Exploring context dependency in eco‐evolutionary patterns with the stick insect *Timema cristinae*

**DOI:** 10.1002/ece3.6526

**Published:** 2020-07-15

**Authors:** Gabriela Montejo‐Kovacevich, Timothy Farkas, Andrew Beckerman, Patrik Nosil

**Affiliations:** ^1^ Department of Zoology University of Cambridge Cambridge UK; ^2^ Department of Biology University of New Mexico Albuquerque NM USA; ^3^ Department of Animal and Plant Sciences University of Sheffield Sheffield UK

**Keywords:** camouflage, eco‐evolutionary dynamics, maladaptation, metacommunity

## Abstract

Rapid evolution can influence the ecology of populations, communities, and ecosystems, but the importance of evolution for ecological dynamics remains unclear, largely because the contexts in which evolution is powerful are poorly resolved. Here, we carry out a large observational study to test hypotheses about context dependency of eco‐evolutionary patterns previously identified on the stick insect *Timema cristinae*. Experiments and observations conducted in 2011 and 2012 documented predator‐mediated negative effects of camouflage maladaptation (i.e., evolutionary dynamics) on: (a) *T. cristinae* abundance and, (b) species richness and abundance of other arthropods. Here we show that camouflage maladaptation does not correlate with *T. cristinae* abundance and, instead, is associated with increased abundance and species richness of cohabitating arthropods. We furthermore find that plants with high levels of *Timema* maladaptation tend to have higher foliar nitrogen, that is, higher nutritional value, and more positive mass‐abundance slopes in the coexisting arthropod communities. We propose explanations for the observed contrasting results, such as negative density‐ and frequency‐dependent selection, feedbacks between herbivore abundance and plant nutritional quality, and common effects of predation pressure on selection and prey abundance. Our results demonstrate the utility of observational studies to assess the context dependency of eco‐evolutionary dynamics patterns and provide testable hypotheses for future work.

## Graphical abstract

Rapid evolution can influence the ecology of populations, communities, and ecosystems, but the importance of evolution for ecological dynamics remains unclear, largely because the contexts in which evolution is powerful are poorly resolved. Here, we synthesize prior research on the stick insect *Timema cristinae* with a new observational study to test hypotheses about context‐dependency. Our results demonstrate the utility of studies focused on context‐dependency in eco‐evolutionary dynamics and provide testable hypotheses for future work.

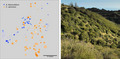

## 1 INTRODUCTION

Research into reciprocal interactions between rapid evolution and ecological dynamics, so‐called “eco‐evolutionary dynamics,” makes clear that rapid evolution can have strong influences on important properties of ecological systems. Numerous studies have shown rapid evolution to influence (meta)population dynamics (Farkas, Mononen, Comeault, Hanski, & Nosil, 2013; Farkas, Mononen, Comeault, & Nosil, 2016; Moore & Hendry, 2009; Ozgul et al., 2010; Turcotte, Reznick, & Hare, 2011), community structure (Brunner, Anaya‐Rojas, Matthews, & Eizaguirre, 2017; Farkas et al., 2013; Johnson, Vellend, & Stinchcombe, 2009; Urban, 2013), and ecosystem functioning (Bassar et al., 2010; Harmon et al., 2009; Matthews, Aebischer, Sullam, Lundsgaard‐Hansen, & Seehausen, 2016; Simon et al., 2017). There is even evidence for long‐hypothesized eco‐evolutionary feedback loops (Brunner et al., 2017; Farkas & Montejo‐Kovacevich, 2014; Matthews et al., 2016; Yoshida, Jones, Ellner, Fussmann, & Hairston, 2003), though most evidence stems from lab experiments or semi‐natural mesocosms, rather than from the wild (Hendry, 2016). In light of this evidence, eco‐evolutionary research shows clear promise to improve the predictive strength of ecological models. But integrating evolutionary dynamics into predictive ecological science requires a precise knowledge of context dependency that is presently too incomplete to be of general use to practitioners. Specifically, flexible models will require parameterization allowing various contexts to modify the strength of eco‐evolutionary dynamics: when, where, and for whom (if ever) will evolution will be a powerful driver of contemporary ecological change in nature?

Although an empirical program to resolve context dependency would be ideal, some inferences can be made on first principles that help to ensure some generality in application. First, evolutionary biology has developed a rich knowledge of when species might undergo evolution rapidly enough to affect ecological patterns we care about. According to the Breeder's Equation, for example, evolution by natural selection will proceed most rapidly when selection is strong and additive genetic variance for selected traits is high (Fisher, 1930; Lande, 1979), and adaptation in absolute time will occur more rapidly if generation times are short. Evolution by gene flow will occur most rapidly when dispersal is high, and when spatial heterogeneity leads to genetic disparity among local populations across a landscape (Slatkin, 1985). Evolution by random drift is more potent in small populations. Thus, study systems with strongly selected traits whose populations are small and inhabit a heterogenous selective landscape are good candidates for detecting rapid evolution that could affect ecological dynamics.

Ecology offers further generalizations (Hendry, 2016). For effects on the population dynamics, the evolution of traits with large effects on fitness should have the largest impact (Bailey et al., 2009; Saccheri & Hanski, 2006). For community structure, the rapid evolution of traits that most influence species interactions will lead to larger ecological effects, and species that engage in many, strong interactions (e.g., keystone species) and/or are particularly abundant may be most likely to have large eco‐evolutionary effects on communities (Bailey et al., 2009; Farkas, Hendry, Nosil, & Beckerman, 2015; Whitham et al., 2006). Similar conclusions can be drawn about the effects of evolution on ecosystem function; traits directly influencing ecosystem properties, for example, the strength of mutualism between plants and nitrogen‐fixing bacteria, may be most impactful (Bailey et al., 2009; Van Nuland, Bailey, & Schweitzer, 2017; Whitham et al., 2006).

Conceptual generalizations like these help to form a strong foundation for predictive eco‐evolutionary science, but focused empirical research is needed to evaluate these generalizations, and ultimately to parameterize models spanning a range of system specificity. Two general approaches can be used. First, experiments with factorial manipulations can help to uncover the conditions under which evolution is a powerful driver of ecology (Bassar et al., 2015; Brunner et al., 2017; Farkas et al., 2013). For example, Brunner et al. (2017) demonstrated that eco‐evolutionary feedbacks between stickleback ecotype divergence and ecological conditions in aquatic mesocosms existed only under specific combinations of ectoparasite load and nutrient addition. Likewise, Turcotte et al. (2011) found effects of rapid evolution on population growth in green peach aphids only when resources were scarce due to browsing herbivory. Second, repeated investigation of eco‐evolutionary systems in natural contexts may demonstrate effects of rapid evolution that vary in strength or pattern across space and time. Such variation provides the opportunity to develop hypotheses and experiments to explain patterns. In this study, we use the second approach, reporting results from an observational field study of eco‐evolutionary patterns in the stick insect *Timema cristinae* that contrast with the results of prior empirical studies. Based on the results, we develop hypotheses that could explain the discrepancies and be tested in future studies.

Previous work on the eco‐evolutionary dynamics of *T. cristinae* demonstrate that camouflage maladaptation strongly influences the structure of their metapopulations and the coexisting plant‐arthropod community of which they are a part (Farkas et al., 2013). Here, maladaptation is defined as the proportion of a local *T. cristinae* population that is composed of the less camouflaged of two color‐pattern morphs (striped vs. unstriped), given the host‐plant species on which morphs live (*Adenostoma fasciculatum* and *Ceanothus spinosus*). While natural selection from avian predators works to create locally adapted populations with good camouflage (Gompert et al., 2014; Nosil & Crespi, 2006), dispersal throughout the landscape works to break down local adaptation through gene flow. This leads to a geographic mosaic of (mal)adaptation, which depends largely on the spatial configuration of the two host‐plant species in the landscape (Bolnick & Nosil, 2007; Sandoval, 1994a, 1994b).

Here, we present a detailed observational study on relationships between camouflage maladaptation and *T. cristinae* abundance, arthropod abundance, and arthropod species richness, where the data were collected one to two years after the above‐mentioned work. To help develop and test mechanistic hypotheses about top‐down and bottom‐up drivers of community structure, we additionally evaluate mass‐abundance relationships in local arthropod communities and foliar carbon/nitrogen content of the plants on which these communities live. Mass‐abundance relationships help define community structure linked to the strength of top‐down predation and energy flow (e.g., Cohen, Schittler, Raffaelli, & Reuman, 2009; Navarrete & Menge, 1997). Understanding foliar nitrogen content is useful because it typically has positive effects on herbivore populations (Throop & Lerdau, 2004). Based on previous studies, we expected negative correlations between maladaptation and *T. cristinae* abundance, arthropod abundance, and arthropod species richness. We also expected that the slope of mass‐abundance relationships for local arthropod communities would negatively correlate with maladaptation, as increased predation pressure where *T. cristinae* are more poorly camouflaged should disproportionately affect larger arthropod prey, decreasing mass‐abundance slopes. Finally, we predicted that foliar carbon:nitrogen ratios would negatively correlate with herbivore abundance due to positive, bottom‐up effect of nitrogen on herbivore populations.

These expectations were not met. Instead, we found that maladaptation did not correlate with *T. cristinae* abundance, correlated positively with non‐*Timema* arthropod abundance, biodiversity, and mass‐abundance slopes, and correlated negatively with foliar carbon:nitrogen ratios. We demonstrate these effects in the Results section and then integrate them in the Discussion to explore mechanistic hypotheses that may underlie them, including negative density‐ and frequency‐dependent natural selection, feedbacks between plant quality and arthropod abundance, and natural variation in predation pressure.

## 2 METHODS

### 2.1 Study system

*Timema cristinae* is a flightless, univoltine stick insect endemic to a small region of chaparral near Santa Barbara, California, where it is the only resident member of the genus *Timema* (Sandoval, 1994a). *Timema cristinae* is folivorous and feeds predominantly on chamise (*Adenostoma fasciculatum*; Rosaceae) and *Ceanothus spinosus* (*Rhamnaceae*). Different color‐pattern morphs have evolved through divergent natural selection by avian predators to achieve good camouflage on these two host‐plant species. “Striped” individuals are green with a single white stripe running longitudinally on the dorsum and are better camouflaged on *Adenostoma*, whereas “unstriped” individuals lack this stripe and are better camouflaged on *Ceanothus* (Bolnick & Nosil, 2007; Sandoval, 1994a, 1994b). Rarer “melanic” forms are brown to red in color, do not express a stripe, and do not demonstrate differential fitness across host‐plant species (Comeault et al., 2015; Lindtke et al., 2017; Nosil et al., 2018).

The community of arthropods living with *T. cristinae* is abundant and diverse, hosting at least 150 morphospecies (Farkas et al., 2013), and *T. cristinae* is often the dominant herbivore, regularly equaling the density of all lepidopteran larvae combined. This makes *T. cristinae* an excellent model for understanding the ecological effects of evolution, since their relatively high abundance makes them a potentially influential member of the community and its dynamics.

### 2.2 Study site

**Figure 1:**
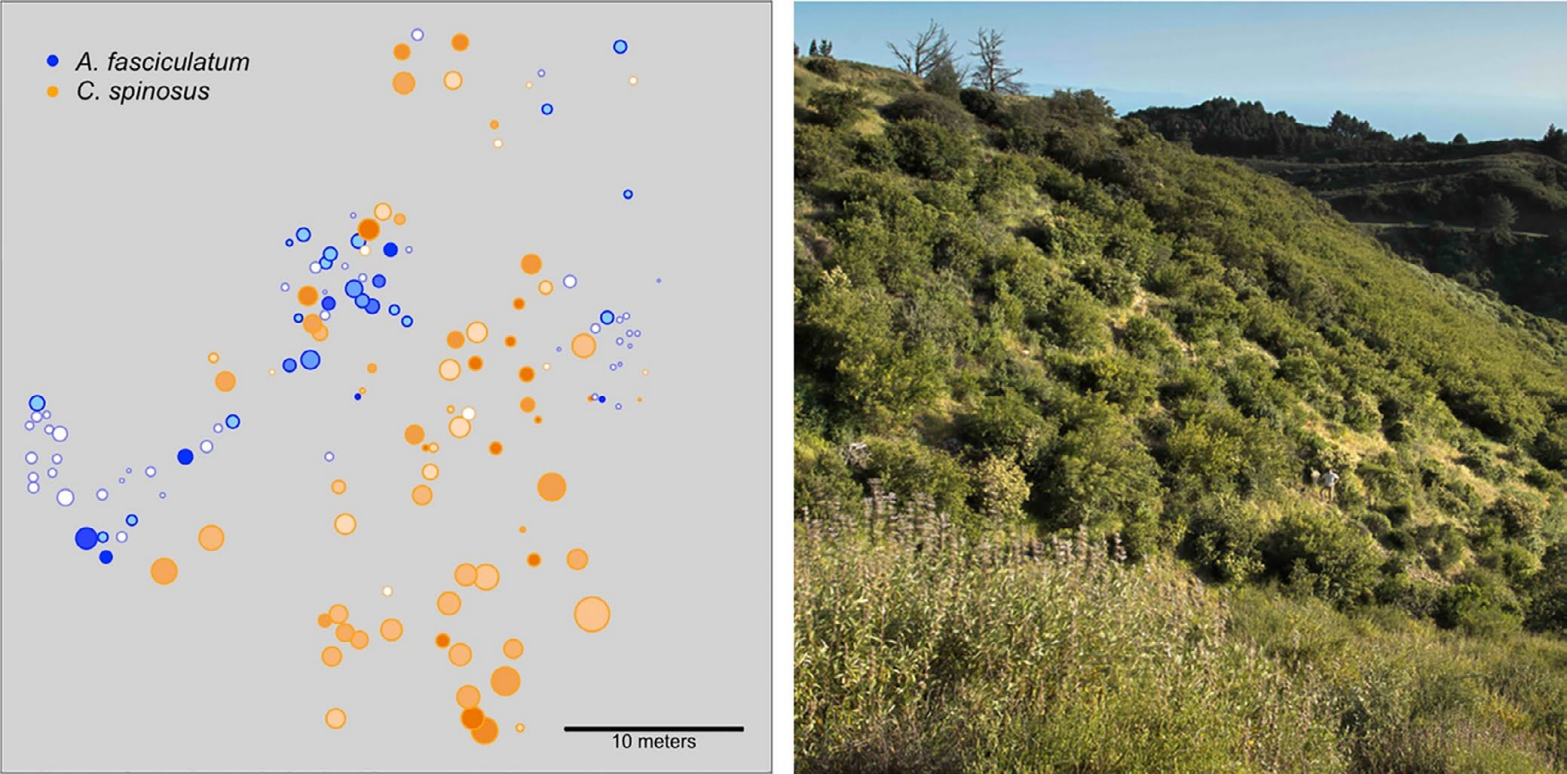
Map of host‐plant patches (left) and picture of part of the network (right). Size of circles is 2‐dimensional projection of rectangular‐solid plant patch volume. Darkness of symbol‐fill color represents degree of maladaptation, where dark indicates more poorly camouflaged populations. Unfilled, semi‐transparent white circles represent patches with no sampled *Timema cristinae*, and border color represents plant species

We selected a 70‐by‐50 m area of chaparral located no >1 km from the experimental blocks conducted in 2011 and 2012 (Figure 1; 34.517N, −119.796W). The area contained an even mixture of *A. fasciculatum* and *C. spinosus* bushes (both species *n* = 73) was situated on a moderate slope and had a north‐westerly aspect. This network of host‐plants will hereafter be referred to as the “network” (Figure 1). To the southwest edge of the network existed a large (~50 m × 100 m) patch of *C. spinosus*, and to the northwest existed a dry, sparse landscape dominated by *A. fasciculatum*. Regions to the north and west, near to the network, were not densely populated by woody perennials of any kind.

### 2.3 Sampling protocol

Between 15 and 19 April 2013, we sampled all host‐plant patches (i.e., individual plants) in the network for arthropods and *Timema* by vigorously shaking all branches and catching fallen individuals in a sweep net (Bioquip). The sweep net was then emptied into a large (~90 cm × 45 cm × 30 cm) plastic tub. In order to ensure collection of all individuals ≥5 mm in length, all individuals determined by eye to be ≥2 mm were collected using soft forceps or aspirator (Bioquip) and preserved in 70% EtOH. Each plant individual was marked with unique identification number from 001 to 147 on an aluminum tag attached to a prominent branch.

On 22 and 23 April 2013, we measured the dimensions of all sampled host‐plants by measuring longest distance across the plant parallel to the ground (length), longest distance across the plant perpendicular to the length (width), and longest distance across the plant perpendicular to the ground (height). At the time of measuring plant dimensions, we also determined the geographic location of each plant to 10 cm accuracy using a GeoExplorer XH GPS (Trimble) with differential correction.

### 2.4 Sample processing

All individual arthropods and *Timema* were photographed under a stereomicroscope with a printed scale‐bar, and we measured body length of each individual from photographs with ImageJ image processing software. A synoptic collection was curated combining samples from prior years’ study in addition to the samples from the current study, and 149 morphospecies were defined. Each morphospecies was identified to the finest taxonomic resolution possible (usually to family) with the available resources (Johnson & Triplehorn, 2004), and each arthropod was assigned to one of the morphospecies using the photographic collection. For morphospecies with fewer than fifteen individuals collected in total, we measured the wet mass of each individual on a standard balance. For morphospecies with greater than fifteen individuals, we measured the wet mass of fifteen individuals spanning the range of observed body length. We fit quadratic curves to regressions of body length on biomass for each morphospecies independently and used regression equations to predict body mass from body length for all individuals not weighed.

### 2.5 Community ecology variables

*Timema* abundance, non‐*Timema* arthropod abundance, and arthropod species richness (excluding *T. cristinae*) data were retained as counts in statistical models. Because of evidence that birds are the primary drivers of eco‐evolutionary effects in this system (Farkas et al., 2013), and because birds are known to be size‐selective predators (Remmel, Davison, & Tammaru, 2011), we calculated arthropod abundance as the number of arthropods equal to or greater than 5 mm in body length. Species richness was calculated as the number of morphospecies in a sample having at least one individual ≥5 mm. All *T. cristinae* were >5 mm in body length due to their advanced life stage at the time of sampling, and so all were included in measurements of *Timema* abundance.

### 2.6 Maladaptation and connectivity

Maladaptation was calculated for each *A. fasciculatum* patch as the number of unstriped *Timema* divided by the total number of *Timema,* and for each *C. spinosus* as the number of striped *Timema* divided by the total number of *Timema*. Melanic *Timema* (31 of 421 individuals) were not included in the abundance totals. Recent experimental evidence shows selection for camouflaged individuals on *A. fasciculatum* to be negatively frequency‐dependent, with a selection coefficient of *s* = 0.7 when rare (20% camouflaged) and *s* = 0 when common (80% camouflaged) (Nosil et al., 2018). Thus, morph frequency may not be the most accurate measure of bird predation intensity. We cannot with currently available data improve our measure of predation intensity, because we do not know the functional form of frequency‐dependence and also do not know whether any function might apply equally to predation on *A. fasciculatum* (where the experiment was conducted) and *C. spinosus*. Therefore, we present in the Appendix 1 parallel analyses to those presented in the main text, using estimates of bird predation intensity assuming a functional relationship between morph frequency and predation intensity ranging from linear to strongly asymptotic (Hughes, 1979; Merilaita, 2006), applying same functional forms equally to both host plants in each case. Results of these analyses mirror those using untransformed morph frequency over a broad range of functional relationships and are not further discussed in the main text.

Demographic connectivity between plant patches of the network was estimated with the following equation (as in Farkas et al., 2013):

$$C_{i}=\sum_{j\neq i}N_{j}\frac{\alpha^{2}}{2\pi }e^{‐\alpha d_{\mathrm{ij}}}$$

where *N_j_* is the number of individuals in source population *j*, and the factor *α* ^2^/2*π* scales the measure so that the integral of the dispersal kernel is one over the two‐dimensional space. The last term is the exponential dispersal kernel, in which *d_ij_* is the distance between plant patches *i* and *j* in meters. The average movement distance is given by 1/*α*, which was assumed to be 2 m, consistent with mark‐recapture estimates of dispersal distances from previous studies (Sandoval, 2000). This equation was used for both *T. cristinae* specifically and for other arthropods, by weighting connectivity calculations with *T. cristinae* and non‐*Timema* abundances, respectively.

### 2.7 Host‐plant variables

Plant volume was calculated as a rectangular solid (*L* × *W* × *H*) and was natural log transformed prior to analysis because volume values spanned multiple orders of magnitude. The sizes of 20 bushes were measured in 2014 due to corrupted electronic data. To estimate the 2013 volume of plants measured only in 2014, we performed a simple linear model predicting 2013 volume with 2014 volume, host‐plant species and their interaction, using data from 10 each of *A. fasciculatum* and *C. spinosus* plants measured in both years. This model showed no significant interaction between 2014 volume and plant species (*p* = .887), suggesting host species did not grow differentially. The model showed a significant effect of 2014 volume (*p* < .001) averaging across plant species, but the intercept (at volume = 0 and averaging across plant species) was not significantly different than 0 (*b* = −1.24 × 10^4^, *p* = .528) and the slope was less than 1 (*b* = 0.88), suggesting that larger plants grew more than smaller plants. At the mean of 2014 volume, host‐plant species was not a significant predictor of 2013 volume (*p* = .731). We then used the parameter estimates from this model to predict 2013 volume using 2014 volume and host‐plant species and used these estimates in all subsequent analyses.

### 2.8 Foliar carbon‐nitrogen assays

Four 4‐inch branch tips were collected haphazardly from each individual host plant and stored in 70% EtOH. We randomly selected five leaves from *Ceanothus* plants and five leaves from each of the four branches of *Adenostoma* plants to pool for [C]:[N] analysis. Leaves were dried at 80°C to constant weight and analyzed for elemental carbon and nitrogen content using an Elementar Vario EL Cube (Elementar Analysengerate GmbH).

### 2.9 Statistical analyses

We modeled natural variation in *Timema* abundance, arthropod abundance, and arthropod species richness (the dependent variables) among plants using generalized linear models with Poisson‐distributed error and quasi‐likelihood estimation, implemented with the glm function in R. For a model of *Timema* abundance, predictor variables initially included maladaptation, host‐plant species, plant volume, carbon:nitrogen ratio, connectivity to *Timema* populations, and all two‐way interactions with host‐plant species. We used backward selection to remove terms according to level of significance, retaining terms at a threshold of *α* = 0.10. Additionally, we excluded all plant patches having fewer than five *Timema* individuals to remove the quadratic relationship between camouflage maladaptation and abundance induced by including samples with few *Timema,* which are more likely to have extreme maladaptation values (Appendix 1). Models of arthropod abundance and species richness were selected as for *Timema* abundance, except connectivity was calculated for arthropod communities (see above). Furthermore, arthropod models included samples with at least one *Timema*, so we included a two‐way interaction term between maladaptation and *Timema* abundance to account for uncertainty in maladaptation values and a hypothesized increase in the importance of *Timema* maladaptation with increasing *Timema* abundance. When the interaction between maladaptation and *Timema* abundance was non‐significant, both the interaction and the main effect of *Timema* abundance were removed from the model.

We characterized the arthropod mass‐abundance size distributions by calculating mass‐abundance slopes on each plant (local community), log‐transforming all arthropod biomass data and dividing the range of log biomass across all plants into twenty, equally sized bins (Gilljam et al., 2011). The abundance of arthropods in each of the twenty bins was calculated for each plant, excluding bins with zero abundance from analysis. We used ordinary least squares regression to relate the natural log of abundance to biomass‐bin rank (1–20) and extracted regression coefficients that represent the slope of the relationship between arthropod biomass and abundance on each host plant. To aid the interpretation of variation in mass‐abundance slopes, we ran one model in which the biomass‐bin rank was centered on the first bin, and another in which rank was centered on the last (20th) bin for downstream analysis. *Timema* were excluded from mass‐abundance analysis. We examined interactions between arthropod communities and their host plants by building models with mass‐abundance slopes and carbon:nitrogen ratios as response variables. These models were selected as for arthropod models above, except we used ordinary least squares regression with the lm function in R, and arthropod abundance was included as a predictor (as well as its interaction with host‐plant species).

In all models, when interaction terms with host‐plant species were significant (at *α* = 0.10), we evaluated parameter estimates for main effects of the interacting term on both hosts independently by running two models with alternate dummy contrasts (0 and 1 vs. 1 and 0). The host‐plant effect was evaluated at the average for all other terms by mean‐centering continuous variables. Controlling for host‐plant species and interactions with the degree of maladaptation is important because the degree of maladaptation on *C. spinosus* was 26.2% higher than on *A. fasciculatum* (*t*_1,91_ = 3.26, *p* = .002), consistent with a broader landscape surrounding the network being dominated by *A. fasciculatum*.

Variation among local communities in the mass‐abundance slope could be due either to differences in the abundance of high‐mass arthropods, low‐mass arthropods, or both. To characterize which of these three scenarios best explained covariation of mass‐abundance slope and maladaptation in each host‐plant species, we evaluated the difference in arthropod abundance between maladaptation extremes at each of the twenty biomass bins. To do this, we use twenty separate models to evaluate the intercepts of mass‐abundance relationships, where biomass data were sequentially centered on each of the twenty biomass bins. We then used ordinary least squares regression with lm in R to relate the mass‐abundance intercepts to maladaptation for each of the twenty intercepts (twenty models total), where a significant effect of maladaptation indicates a difference in abundance for a particular biomass bin.

For main‐effect parameters that were selected out of statistical models, we report *t‐* and *p‐*values from the step in backward selection prior to removing the term. Statistics for nonsignificant interaction terms are not reported. When maladaptation was a significant predictor, we furthermore calculated partial, adjusted r‐square values for all variables in the final model to compare the effect magnitude of maladaptation to ecological drivers. When interactions with host‐plant species were retained in final models, we evaluated r‐square values on the host‐plant species for which significant effects were seen. When original models were generalized linear models with Poisson‐distributed error, we performed an ordinary least squares regression on square‐root transformed count data to achieve partial r‐square values.

## 3 RESULTS

### 3.1 *Timema* abundance

The negative demographic influence of *Timema* maladaptation found in 2011 and 2012 was not corroborated in the present observational study, where the relationship between *Timema* abundance and maladaptation was nonsignificant (*t*_1,34_ = −0.02, *p* = .984). Furthermore, *Timema* abundance did not differ across the two host plants (*t*_1,35_ = 0.29, *p* = .773) nor did it correlate with foliar [C]:[N] ratios (*t*_1,33_ = 0.05, *p* = .96). Consistent with past research (Farkas et al., 2016), *Timema* abundance was positively influenced by connectivity to other *Timema* populations (*t*_1,36_ = 2.30 *p* = .027) and patch volume (*t*_1,36_ = 2.56, *p* = .015), supporting well‐established metapopulation theory (Hanski, 1998). All two‐way interactions with host‐plant species were nonsignificant and hence were selected out of the final model. Connectivity and plant volume together explained 19% of natural variation in *Timema* abundance.

### 3.2 Arthropod abundance

**Figure 2:**
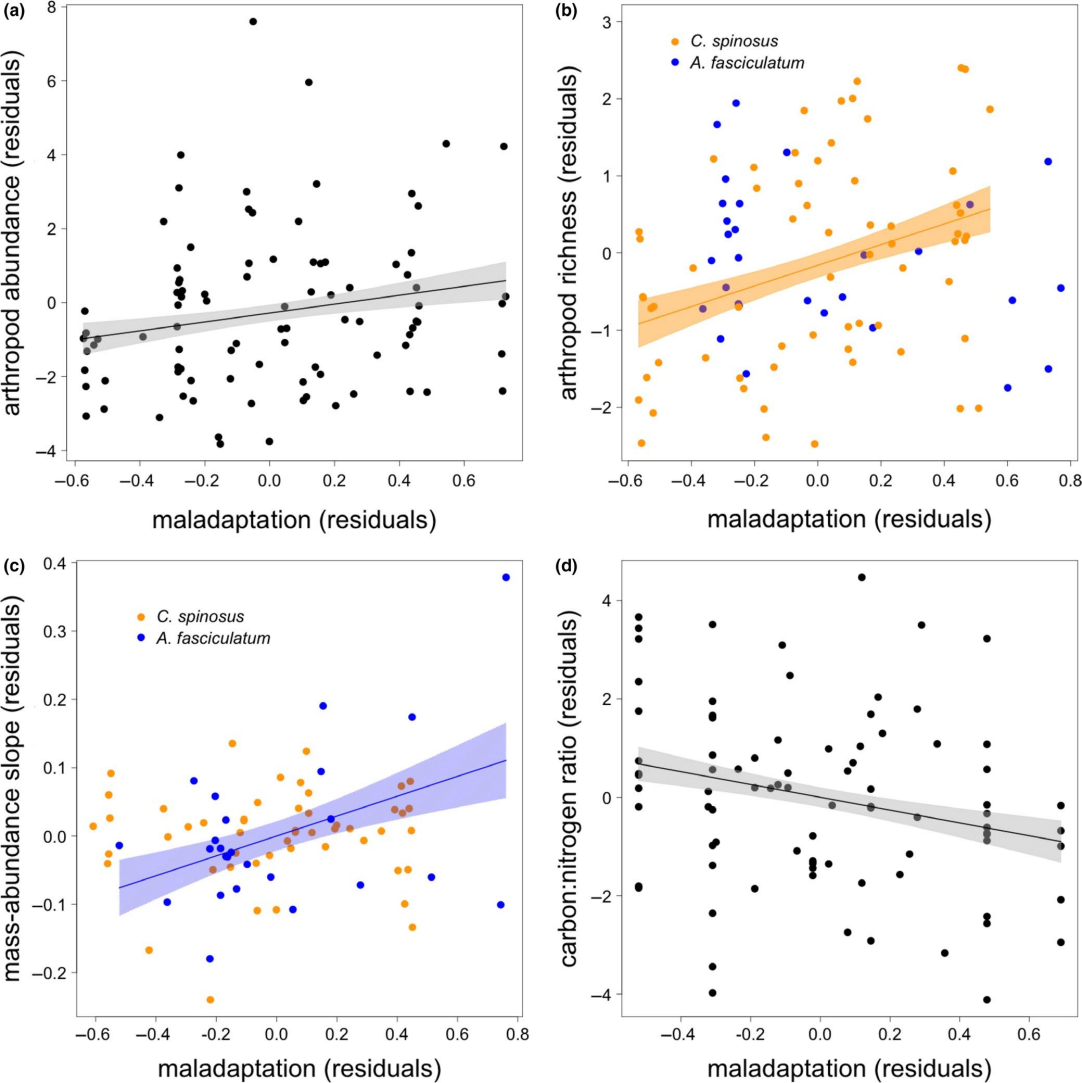
Residual‐residual plots showing effects of maladaptation on (a) arthropod abundance, (b) arthropod species richness, (c) the slope of arthropod mass‐abundance relationships, and (d) foliar carbon:nitrogen ratios. Lines show best linear fit for significant effects and are colored to indicate effects on one host‐plant species only. Shaded areas show confidence bands at 1 *SE* for local communities on *A. fasciculatum* and *C. spinosus* pooled (a, d), communities on *C. spinosus* only (b), and communities on *A. fasciculatum* only (c)

The expected negative influence of *Timema* maladaptation on the abundance of other arthropod species was not supported; instead, patches with higher maladaptation also had higher arthropod abundance (Figure 2a; *b* = 0.50, *t*_1,87_ = 2.11, *p* = .038). As with *Timema* abundance, patch volume positively influenced arthropod abundance (*t* = 5.37, *p* < .001).

Further analyses revealed a number of interactions. The interaction between host‐plant species and assemblage connectivity showed a marginal trend (*t* = 1.82, *p* = .072), and accordingly, there was a significant positive effect of connectivity for assemblages inhabiting *C. spinosus* (*t* = 2.58, *p* = .012), but no significant effect on *A. fasciculatum* (*t* < 0.01, *p* = .997). At average assemblage connectivity, there was significantly higher arthropod abundance on *A. fasciculatum* than on *C. spinosus* (*t* = 2.20, *p* = .030), a pattern that does control for the larger volume of *C. spinosus* plants in this network. There was no relationship between arthropod abundance and [C]:[N] ratios (*t*_1,79_ = −1.15, *p* = .255). Restricting analysis to *C. spinosus* (which showed a significant effect of connectivity), maladaptation, connectivity, and plant volume explained 5.91%, 7.11%, and 33.7% of variation in arthropod abundance, respectively.

### 3.3 Arthropod species richness

Consistent with a strong correlation between arthropod abundance and species richness (Poisson GLM: *t*_1,144_ = 12.49, *p* < .001), results for correlates of species richness largely mirror those on abundance. Novel to this analysis, there is a significant interaction between host‐plant species and maladaptation (Figure 2b; *t* = 2.48, *p* = .015), with a significant positive effect of maladaptation for assemblages inhabiting *C. spinosus* (*t* = 3.40, *p* = .001), but a nonsignificant effect of maladaptation for assemblages inhabiting *A. fasciculatum* (*t* = 0.78, *p* = .440). As with arthropod abundance, there was a significant interaction between assemblage connectivity and host‐plant species (*t* = 2.90, *p* = .005), with a significant positive effect on *C. spinosus* (*t* = 3.04, *p* = .003) and a nonsignificant effect on *A. fasciculatum* (*t* = 1.21, *p* = .229). Patch volume positively correlated with richness (*t*_1,86_ = 6.58, *p* < .001), but richness did not differ between the host‐plant species when maladaptation and connectivity are average (*t* = 0.13, *p* = .89). There was no relationship between arthropod richness and [C]:[N] ratio (*t*_1,78_ = −0.84, *p* = .403). Restricting analysis to *C. spinosus* (where maladaptation and connectivity were significant predictors), maladaptation, connectivity, and plant volume explained 9.92%, 5.84%, and 42.3% of variation in arthropod species richness.

### 3.4 Mass‐abundance relationships

There was a significant positive relationship between *T. cristinae* maladaptation and the slope of mass‐abundance regressions (MAS) on *A. fasciculatum* (Figure 2c; *t* = 3.50, *p* < .001), indicating that the ratio of large to small arthropods increased with maladaptation, but there was no relationship on *C. spinosus* (*t*_1,67_ = 0.32, *p* = .753), consistent with a significant interaction between maladaptation and host‐plant species (*t*_1,67_ = *−2.63*, *p* = .011). Additionally, the effect of maladaptation increased with *Timema* abundance (*t*_1,67_ = 3.46, *p* < .001), supporting a hypothesis of density‐dependence in these eco‐evolutionary effects. Furthermore, there was a negative relationship between arthropod abundance and MAS on both host‐plant species (C: *t*_1,67_ = −2.75, *p* = .007; A: *t*_1,67_ = −3.61, *p* < .001), but the effect was more negative on *A. fasciculatum* (interaction: *t*_1,67_ = −2.02, *p* = .048). MAS was significantly lower on *C. spinosus* (*t*_1,67_ = −2.38, *p* = .020), and there was no relationship between MAS and [C]:[N] ratio (*t*_1,59_ = −0.17, *p* = .868) or plant volume (*t*_1,60_ = 0.94, *p* = .348). On *A. fasciculatum* only (where significant effects of maladaptation were found), maladaptation and arthropod abundance explained 5.91% and 7.01% of variation in MAS, respectively.

**Figure 3:**
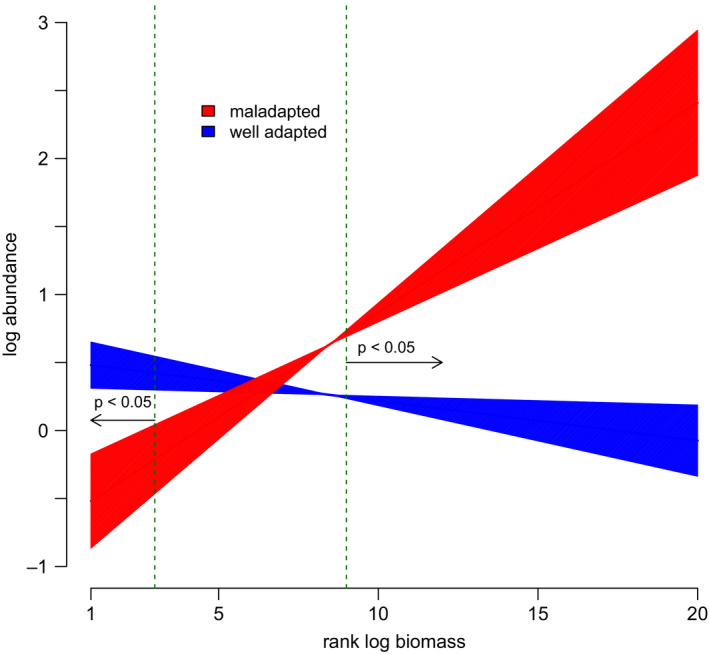
Predicted relationship between log arthropod abundance and log biomass at maladaptation extremes in *A. fasciculatum*. Dashed vertical lines delimit biomass ranges over which there is a significant effect of maladaptation on abundance. Confidence bands show one standard error for intercept estimates

To further investigate the effects of maladaptation on the slopes of mass‐abundance relationships on *A. fasciculatum*, we assessed the difference in arthropod abundance between maladaptation extremes at each of the twenty biomass bins. Interestingly, these predicted MAS to be negative under minimum maladaptation, but positive under maximum maladaptation (Figure 3). This pattern was largely due to increases in the abundance of high‐mass arthropods, though significant decreases in low‐mass arthropods were also found. Arthropod abundance was significantly lower when *T. cristinae* were maximally maladapted for biomass bins 1–3, and significantly higher for bins 9–20 (Figure 3).

### 3.5 Foliar Carbon‐Nitrogen Composition

**Figure 4:**
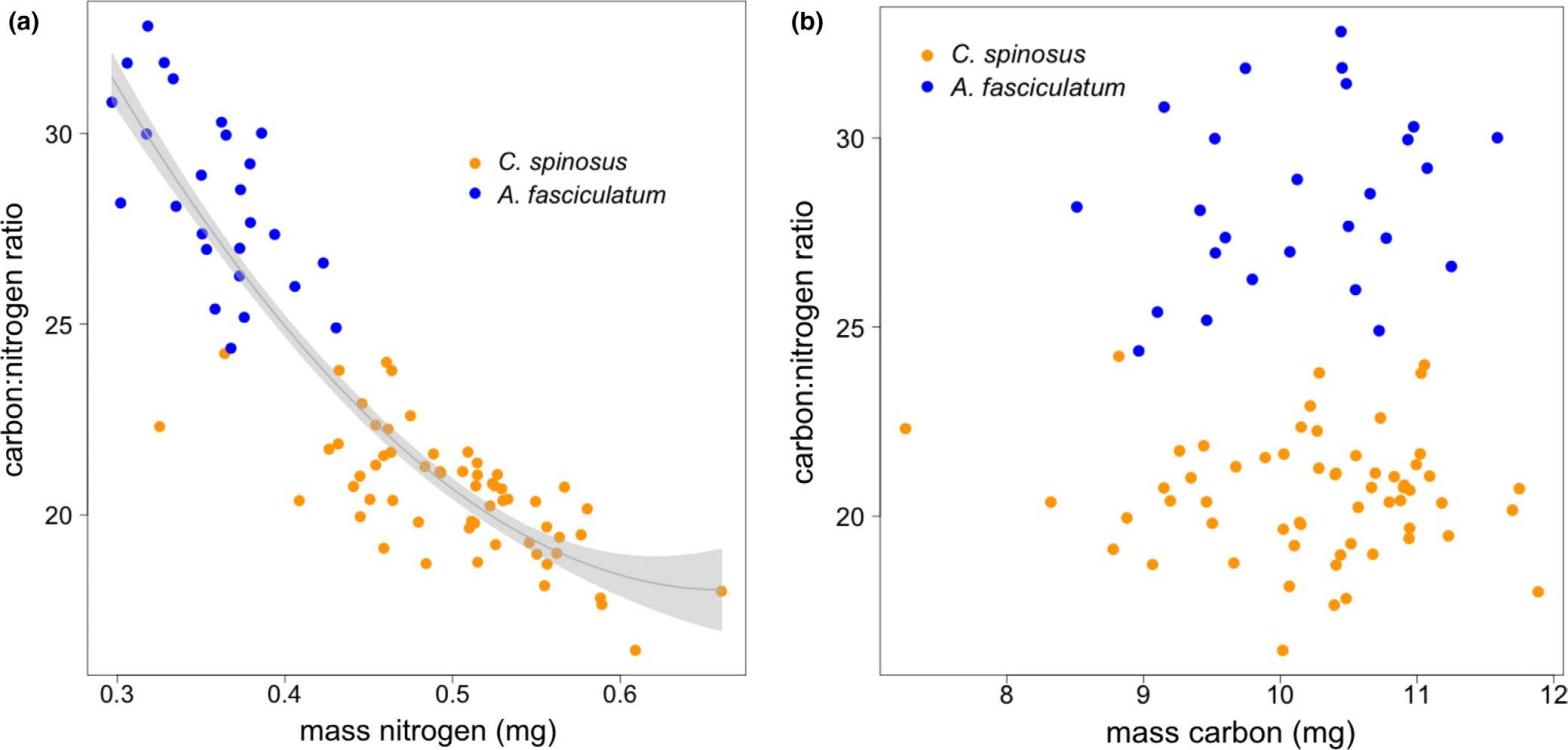
Relationship between carbon:nitrogen ratio and (a) mass nitrogen, (b) mass carbon. Gray line in (a) shows best fit quadratic curve and shaded area shows confidence bands at ±1 *SE*

*Ceanothus* plants had significantly lower [C]:[N] than *Adenostoma* plants (*t*_1,83_ = −17.13, *p* < .001). We found a significant negative relationship between maladaptation and [C]:[N] (Figure 2d; *t*_1,83_ = −2.24, *p* = .028), but [C]:[N] ratios were correlated with neither plant volume (*t*_1,81_ = 1.10, *p* = .275) nor arthropod abundance (*t*_1,82_ = −1.19, *p* = .237). Variation in [C]:[N] across plant patches was largely driven by variation in nitrogen rather than carbon (Figure 4; [C]:[N] ~ [N]: *p* < .001; [C]:[N] ~ [C]: *p* = .39), such that lower [C]:[N] on *Adenostoma* plants and on plants with more poorly camouflaged *T. cristinae* largely indicate higher concentrations of nitrogen (Figure 4a), rather than lower concentrations of carbon (Figure 4b).

## 4 DISCUSSION

Two manipulative field experiments and one observational study showed that poor camouflage leads to reduced stick insect population size due to intensified bird predation (Farkas et al., 2013) and can even lead to a decrease in habitat patch occupancy (i.e., local extinction) throughout a wild metapopulation (Farkas et al., 2016). These studies also demonstrated that intensified bird predation due to poor camouflage leads to reduced arthropod abundance and species richness, as well as reduced herbivory from sap‐feeding insects. Thus, our expectation for the present study was that maladaptation in *T. cristinae* would lead to lower *T. cristinae* abundance, with cascading negative effects on the abundance and species richness of cohabitating arthropods (Farkas et al., 2013, 2016). We furthermore predicted that mass‐abundance relationships would be more negative on plants with maladapted *T. cristinae* due to an increased predation from positively size‐selective predators (Navarrete & Menge, 1997; Remmel et al., 2011).

In contrast to these predictions, we found that camouflage maladaptation correlated positively with arthropod abundance and species richness (Figure 2a,b) and did not correlate with *T. cristinae* abundance. In addition, we found that in patches with higher *T. cristinae* maladaptation there were disproportionally larger arthropods than in those plants with well‐camouflaged *Timema*, leading to positive correlations between maladaptation and mass‐abundance slopes (Figure 2c). Lastly, we found that leaves tend to be more nitrogenous, that is, of higher nutritional value, where *Timema* are more poorly camouflaged (Figure 2d).

These findings raise the questions of (a) what eco‐evolutionary mechanisms could lead to the specific patterns found here, and (b) what accounts for the observed differences between studies? We offer and discuss four non‐mutually exclusive hypotheses about the contrasting results noted above, and thus context dependency in eco‐evolutionary dynamics. A general theme that emerges is the importance of spatiotemporal variation in the relative strength of various ecological and evolutionary processes, each having either positive or negative influences on eco‐evolutionary properties of local populations and communities. Future experimental work testing these hypotheses is warranted.

### 4.1 Density‐ and frequency‐dependent selection hypotheses

An experimental field study in this system (Farkas & Montejo‐Kovacevich, 2014) demonstrated an eco‐evolutionary feedback loop whereby selection against poorly camouflaged *T. cristinae* is negatively density‐dependent, likely due to predator satiation at high density. Hence, we hypothesize that negatively density‐dependent selection could be the cause of positive relationships between maladaptation and arthropod abundance/diversity in the present study. Under this hypothesis, local communities with high abundance of prey lead predators to satiate quickly, which reduces the strength of selection on *T. cristinae* populations, which, in turn, leads to higher levels of maladaptation. Predictions testable with our data are difficult to construct under this hypothesis. Here, we would predict mass‐abundance slopes to be higher where arthropod abundance is high, because scenarios with satiated predators will have more large prey than scenarios where predators continue to forage, while smaller arthropods will be less affected. We found the opposite pattern, where arthropod abundance correlated negatively with mass‐abundance slopes. We argue, however, that this pattern fails to strongly falsify the density‐dependent selection hypothesis, since increased arthropod abundance could be predicted to reduce mass‐abundance slopes under a neutral mechanism, whereby the addition of individuals at random from the regional pool would add more small than large‐bodied arthropods. Hence, the relative magnitude of predator‐mediated and neutral mechanisms, which is not measurable here, could lead to either a net positive or negative effect of arthropod abundance on mass‐abundance slopes.

Another study in this system demonstrates selection on striped and unstriped morphs in *T. cristinae* to be negatively frequency‐dependent, such that cryptic morphs enjoy an outsized advantage when rare, but no advantage when common (Nosil et al., 2018). Furthermore, fluctuations in morph frequency over long time‐scales (25 years) are highly predictable and conform to a model of negatively frequency‐dependent selection (Nosil et al., 2018). Hence, it may be that selection was particularly weak in the year of this study, especially on *Ceanothus* plants (where positive relationships with maladaptation were strongest), allowing other eco‐evolutionary mechanisms to dominate, leading to patterns that violate predictions based on selection alone. Whether frequency‐dependent selection shows similar patterns across both host‐plant species remains to be tested empirically (Nosil et al., 2018).

### 4.2 Plant‐quality hypothesis

A third hypothesis is based on feedbacks between herbivore abundance and plant quality that lead to temporally fluctuating dynamics. Under this hypothesis, maladaptation in *T. cristinae* does lead to increased predation and reduced arthropod abundance in a given year, but reduced abundance consequently leads to increased plant nutritional quality in later years via lower herbivory (Schultz, Appel, Ferrieri, & Arnold, 2013). Increased plant quality therefore drives increased arthropod abundance from the bottom‐up, but levels of maladaptation remain fairly constant from year to year, since maladaptation itself is driven by gene flow as determined by the spatial configuration of host‐plant species in the landscape (Bolnick & Nosil, 2007; Sandoval, 1994a). The positive relationship between maladaptation and arthropod abundance results when the bottom‐up effects of increased plant quality outweigh the top‐down effects of increased bird predation and predicts that increased foliar nitrogen content leads to higher arthropod abundance, which is generally supported by plant‐herbivore studies in other systems (Throop & Lerdau, 2004).

However, evidence for this hypothesis from the present study is not strong because, although we found a significant positive correlation between maladaptation and foliar carbon:nitrogen ratios in the predicted direction (Figure 2d), we did not find a significant relationship between carbon:nitrogen ratios and arthropod abundance. Hence, we fail to support the full feedback from maladaptation to increased arthropod abundance through plant quality, but we do show some support for the hypothesis that reduced herbivory on plants with high camouflage maladaptation influences foliar elemental composition. A further consideration is that gross carbon and nitrogen content are relatively uninformative due to the complex chemical physiology of plants, where both nitrogen and carbon can be employed toward primary physiological processes required for photosynthesis, or toward constitutive or induced defenses against herbivores and pathogens (Schultz et al., 2013). Further work in this system will use the tools of chemical ecology to resolve more precisely the foliar chemical content and its likely function with respect to plant quality for herbivores.

### 4.3 Variation in bird predation hypothesis

Finally, we propose a hypothesis whereby an external driver of spatial variation in predation pressure simultaneously influences arthropod abundance/diversity and maladaptation. Under this hypothesis, high bird predation leads simultaneously to reduced maladaptation (by selecting poorly camouflaged individuals out of the populations) and reduced arthropod abundance. Consistent with this hypothesis is the finding that mass‐abundance relationships increase with maladaptation, which we predict to result from reduced attack by positively size‐selective predators (Navarrete & Menge, 1997; Remmel et al., 2011). It is difficult to test this hypothesis with the data collected in this study, since we have no direct observation of predation pressure, and have little knowledge of what drives the strength of bird predation outside the previously demonstrated effects of camouflage maladaptation (Farkas et al., 2013). Inconsistent with this hypothesis is the finding that maladaptation does not correlate with the abundance of *T. cristinae*, where we predict a positive relationship.

## 5 CONCLUSIONS

In sum, the observational approach taken here helps to refine and falsify some hypotheses for context dependency in the eco‐evolutionary dynamics of *T. cristinae* and the arthropod community of which it is a dominant member, and furthermore succeeds in motivating focused future experimentation to test viable hypotheses. Accordingly, future work in this system should address nonconsumptive effects of predation risk on community structure, feedbacks between plant chemistry and herbivory, and drivers of predation strength other than the influence of maladaptation. Additionally, a second goal is to understand reasons why particular mechanisms dominate at particular times, so future work will also focus on how the relative strength of these mechanisms fluctuates trough time.

## CONFLICT OF INTEREST

None declared.

## AUTHOR CONTRIBUTION

**Gabriela Montejo‐Kovacevich:** Data curation (equal); Formal analysis (equal); Investigation (equal); Visualization (equal); Writing‐original draft (equal); Writing‐review & editing (equal). **Timothy Farkas:** Conceptualization (equal); Data curation (equal); Formal analysis (equal); Methodology (equal); Project administration (equal); Supervision (equal); Visualization (equal); Writing‐review & editing (equal). **Andrew P Beckerman:** Visualization (equal); Writing‐review & editing (equal). **Patrik Nosil:** Conceptualization (equal); Data curation (equal); Formal analysis (equal); Funding acquisition (lead); Methodology (equal); Project administration (lead); Supervision (lead); Visualization (equal); Writing‐review & editing (equal).

## Figures and Tables

**Figure A1:**
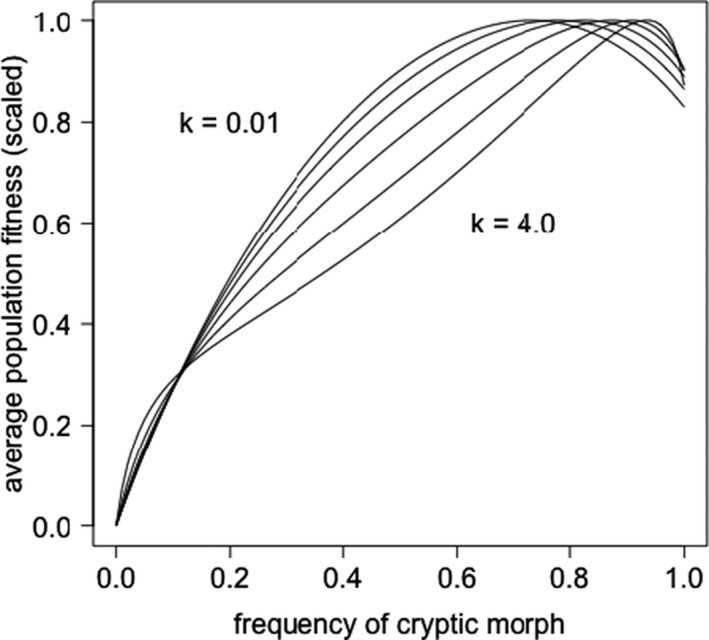
Relationship between the frequency of the cryptic (adapted) morph and scaled average population absolute fitness for *k* = 0.01, 0.6, 1.2, 2.0, 3.0, and 4.0 shown as separate lines

## Data Availability

Data and scripts used for analysis are available on the public repository Zenodo (https://doi.org/10.5281/zenodo.3881476).

## APPENDIX 1

### ESTIMATING FORAGING INTENSITY WITH NEGATIVE FREQUENCY‐DEPENDENT SELECTION

In the main text of this paper and other published studies of eco‐evolutionary dynamics in *T. cristinae*, we use the frequency of the conspicuous morph (maladaptation) as a measure of bird predation strength (equivalently, average absolute fitness of *T. cristinae* populations), under the assumption that the strength of selection for the cryptic morph is constant. However, Nosil et al. (2018) use a field experiment to show negative frequency‐dependent selection (NFDS) for camouflaged *Timema* on *A. fasciculatum*. By transplanting camouflaged (striped) and conspicuous (unstriped) individuals at 1:4 and 4:1 ratios onto wild bushes, they find the stripe to be strongly favored when rare at 1:4 (*s* = 0.70) and neutral when common at 4:1 (*s* = −0.04). Therefore, the magnitude of variation in predation strength with morph frequency should itself vary across the range of morph frequencies. With selection evaluated only at two morph frequencies, however, the functional form of relationship between morph frequency and selection is free to vary widely. Furthermore, whether frequency dependent selection occurs at all on *C. spinosus* is unknown, let alone the functional form of NFDS. Hence, we here explore the consequences for our findings of incorporating NFDS into metrics of bird predation strength to replace the frequency of conspicuous morphs (maladaptation) as presented in the main text.

We follow fundamental theory about consumer learning in optimal foraging to construct NFDS functions ranging from linear to highly asymptotic (Hughes, 1979; Merilaita, 2006) and thereby calculate alternate metrics of average absolute fitness for *Timema* populations. To do this, we fit the selection data from Nosil et al. (2018) to the following asymptotic model developed for relating predator handling time (*I*) to the encounter rate (*λ*) of two prey types (Hughes, 1979):

$$I=\frac{I_{\infty }}{1‐\left(\frac{I_{0}‐I_{\infty }}{I_{0}}\right)e^{‐k\lambda }}$$

*I* represents absolute fitness for a morph, *I*_0_ represents the maximum absolute fitness of a morph (i.e., without predator learning), *I*_∞_ represents the minimum absolute fitness of a morph (i.e., after the predator has optimized search/handling time), and *k* represents the instantaneous rate of change in *I* with respect to encounter rate (*λ*). For our purposes, we equate *λ* with morph frequency. We fit the model to the two data points by minimizing the sum of squares, estimating the values of *I*_0_ *and I*_∞_ for set values of *k* = (0.01, 0.6, 1.5, 2.0, 3.0, 4.0). With *k* = 0.01, the fit approximates linear, where increasing *k* leads to stronger of curvature for an asymptotic fit. To calculate average absolute fitness for a population, we calculate the absolute fitness of each morph with the above equation, multiply fitness by morph frequency, and add the morph‐specific fitnesses. We then scale the result between 0 and 1 to be consistent with the metric of maladaptation presented in the main text. When the form of NFDS is linear, the relationship between cryptic morph frequency and average fitness is quadratic with a peak at 65% cryptic, but with increasing *k* becomes more linear, especially at intermediate morph frequencies, and is accompanied by a shift in maximum fitness toward 100% cryptic (Figure A1).

### METHODS

To test the effect of incorporating NFDS into average absolute fitness, we calculated NFDS‐adjusted maladaptation as (1—absolute fitness) based on the function shown above (Figure A1). We refit the models described in the main text for *Timema* abundance, arthropod abundance, arthropod species richness, mass‐abundance slopes, and carbon:nitrogen ratio, once for each of *k* = 0.01, *k* = 0.6, *k* = 1.2, *k* = 2.0, *k = *3, and *k = *4. We report below the parameter estimates and *p*‐values for NFDS‐adjusted maladaptation and its interaction with host where appropriate (Table A1).

### RESULTS

Compared with the analysis in the main text (Table A1: “No NFDS Transformation”), models using estimates of predation strength that incorporate NFDS were broadly similar. These analyses showed strong trends (*p* < .10) or significant relationships (*p* < .05) between NFDS‐adjusted maladaptation and each of the five response variables (Table A1). The strength of these trends increased with *k* (i.e., an increase the asymptotic nature of the NFDS function), likely due to the increase in linearity of relationship between maladapted morph frequency and average absolute fitness for a population (Figure A1).

**Table A1:** Parameter estimates (*b*) and *p*‐values (*p*) for five models parallel to those shown in main text (reproduced here: “No NFDS Transformation”) using estimate of population fitness incorporating negative frequency‐dependent selection with function weakly (*k* = 0.01) to strongly (*k* = 4) asymptotic

	No NFDS		*k* = 0.01		*k* = 0.6		*k* = 1.2		*k* = 2		*k* = 4	
	*b*	*p*	*b*	*p*	*b*	*p*	*b*	*p*	*b*	*p*	*b*	*p*
*Timema* abundance												
mal	0.02	.931	−0.03	.929	−0.02	.953	−0.01	.978	0.00	.99	0.02	.950
Arthropod abundance												
mal	0.50	**.038**	0.39	.099	0.41	.083	0.43	.070	0.46	.057	0.49	**.047**
Arthropod richness												
mal	0.80	**.001**	0.49	**.017**	0.52	**.011**	0.56	**.007**	0.61	**.004**	0.68	**.003**
mal * host	−1.10	**.009**	−0.76	.108	−0.80	.084	−0.85	.065	−0.92	**.046**	−1.00	**.031**
Mass‐abundance slopes												
mal	0.18	**.001**	0.16	**.011**	0.17	**.007**	0.18	**.004**	0.18	**.002**	0.19	**.001**
mal * host	−0.17	**.009**	−0.13	.058	−0.14	**.040**	−0.15	**.029**	−0.16	**.021**	−0.17	**.016**
Carbon:Nitrogen ratio												
mal	−1.13	**.043**	−1.02	.085	−1.05	.072	−1.09	.062	−1.15	.052	−1.22	**.044**

Note

*p*‐values significant at *α* = 0.05 are highlighted in bold.
